# Beyond Clinicopathological Criteria: A Practical Management Framework for Oral Lichen Planus

**DOI:** 10.3390/medsci14020252

**Published:** 2026-05-13

**Authors:** Doina Iulia Rotaru, Ovidiu Păstrav, Sorana D. Bolboacă, Camelia Lazăr, Radu Marcel Chisnoiu

**Affiliations:** 1 Department of Odontology, Endodontics, and Oral Pathology, Faculty of Dental Medicine, “Iuliu Hațieganu” University of Medicine and Pharmacy, 33 Moților Str., 400001 Cluj-Napoca, Romania; doina.rotaru@umfcluj.ro (D.I.R.); ovidiu.pastrav@elearn.umfcluj.ro (O.P.); marcel.chisnoiu@umfcluj.ro (R.M.C.); 2 Department of Medical Informatics and Biostatistics, Faculty of Medicine, “Iuliu Hațieganu” University of Medicine and Pharmacy, 6 Louis Pasteur Str., 400349 Cluj-Napoca, Romania; 3 CMMD Lazar A. Camelia Augusta, 3 Clujului Str., 405300 Gherla, Romania; cameliaaugusta@yahoo.com

**Keywords:** oral lichen planus (OLP), differential diagnosis, malignant transformation, oral white lesions (OWL), case illustration

## Abstract

*Background*: Oral lichen planus (OLP) is a chronic T-cell-mediated inflammatory disorder classified by the World Health Organization as a potentially malignant disorder. Diagnosis remains challenging due to clinical and histopathological overlap with other oral white lesions, including lichenoid reactions, frictional keratosis, and malignancy. *Objectives*: This systematic search with narrative review aimed to synthesize current diagnostic criteria, characterize key differential diagnoses, and provide an evidence-based diagnostic framework for clinicians. Methods: A comprehensive literature search was conducted across PubMed/MEDLINE, Scopus, Web of Science, and Embase through December 2025. Following a systematic screening process, eligible manuscripts were narratively summarized and a clinical case illustration was demonstrated. *Results*: Twenty-nine of 214 peer-reviewed studies (including systematic reviews, guidelines, and cohort studies) were summarized. Diagnostic standards have evolved toward the American Academy of Oral & Maxillofacial Pathology (AAOMP) 2016 criteria, which emphasize mandatory clinicopathological associations. Key differential diagnoses include reactive lesions (frictional keratosis), infectious conditions (chronic hyperplastic candidiasis), and other lichenoid patterns. Malignant transformation rates are approximately 1.43%, increasing to 5.13% in the presence of dysplasia, necessitating long-term surveillance. An 81-year-old case exemplifies the value of a stepwise diagnostic approach, in which initial management focuses on the elimination of local irritants and a period of clinical observation, followed by histopathological confirmation of oral lichen planus through biopsy when necessary. *Conclusions*: Accurate OLP diagnosis requires integrating clinical presentation with histopathological findings. A systematic diagnostic algorithm—incorporating local factor elimination, selective biopsy, and long-term monitoring—is essential to distinguish OLP from its mimics and manage the risk of malignant transformation effectively.

## 1. Introduction

White lesions account for approximately 5% of all oral mucosal pathology, encompassing conditions ranging from benign reactive changes to potentially malignant disorders and invasive malignancies [1,2]. White lesions often share similar clinical features and may be asymptomatic or cause pain and functional impairment, posing significant diagnostic challenges.

Oral lichen planus (OLP) is a chronic T-cell-mediated inflammatory disorder with a global prevalence of 0.5–2%, predominantly affecting middle-aged and older adults with a female-to-male ratio of approximately 2:1 [1,3,4]. Unlike cutaneous lichen planus, oral lesions rarely resolve spontaneously and may persist for decades. The etiology involves dysregulated T-cell-mediated immune responses directed against basal keratinocytes, with well-established associations including hepatitis C virus (HCV) infection, type 2 diabetes mellitus, thyroid dysfunction, dyslipidemia, and psychological stress [5,6]. Medications implicated in oral lichenoid drug reactions—which closely mimic OLP—include antihypertensives, oral hypoglycemic agents, Nonsteroidal Anti-Inflammatory Drugs (NSAIDs), and antimalarials [7].

Clinically, OLP exhibits remarkable polymorphism: reticular (most common, characterized by white lacy Wickham striae), erosive/atrophic (painful erythematous areas), plaque-like (higher malignant risk), papular, and bullous variants [3,8]. The disease characteristically presents bilaterally and symmetrically, most commonly affecting the buccal mucosa (90%), followed by the gingiva, tongue, and palate [7].

The World Health Organization (WHO) classifies OLP as a potentially malignant disorder. Recent meta-analyses report pooled malignant transformation rates of 1.43%, increasing to 5.13% when epithelial dysplasia is present [9,10]. Risk factors include plaque-like subtype, tongue location, erosive/atrophic forms, tobacco and alcohol use, and HCV infection [11,12].

Diagnosis is challenging because clinical and histopathological features overlap with oral lichenoid lesions, lichenoid contact reactions, and lichenoid drug reactions [1,7,13]. Furthermore, OLP is dynamic—lesions may change appearance over time [14]. Diagnostic complexity is compounded in elderly patients with systemic comorbidities, polypharmacy, and local factors such as ill-fitting prostheses, where multiple conditions may coexist. Recognizing these challenges, diagnostic criteria have evolved from WHO 1978 through modified WHO 2003 to the current American Academy of Oral & Maxillofacial Pathology (AAOMP) 2016 position paper [10], which emphasizes clinicopathological associations and continued follow-up [1,9,14]. The current guidelines recommend medical history and clinical examination, followed by targeted investigations (histopathology, immunofluorescence, serology as appropriate), and culminate in longitudinal follow-up and biopsy when new lesions develop or when there is diagnostic uncertainty or signs of malignant transformation risk [7,15]. Physicians need to manage complex scenarios in clinical practice, involving overlapping lesions, local traumatic factors, polypharmacy, and systemic comorbidities, where distinguishing oral lichen planus from its mimics is challenging [16,17]. Moreover, most published reviews address isolated aspects of diagnosis rather than integrating clinical evaluation, differential diagnosis, ancillary testing, and longitudinal observation into a unified diagnostic strategy [18,19]. The main identified gaps are as follow: the differential diagnosis of OLP encompasses heterogeneous conditions (reactive, infectious, autoimmune, premalignant, malignant) that are rarely addressed collectively in systematic reviews; practical diagnostic algorithms require synthesis across evidence types that may not lend themselves to formal meta-analysis; and integration of a clinical case illustration necessitates narrative flexibility while maintaining methodological rigor in evidence gathering.

Our systematic search of scientific literature with narrative review addresses the gaps with objectives to: (1) synthesize current diagnostic criteria; (2) characterize key differential diagnoses with distinguishing features; (3) evaluate ancillary diagnostic tests, particularly direct immunofluorescence; (4) provide an evidence-based diagnostic algorithm; (5) assess malignant transformation risk and surveillance recommendations; and (6) illustrate practical application through a clinical case demonstrating systematic diagnostic reasoning.

## 2. Materials and Methods

### 2.1. Study Design and Rationale

This study was designed as a narrative review, using a PRISMA-inspired methodology [20], combining systematic search strategies with the interpretive flexibility inherent to narrative synthesis.

### 2.2. Information Sources and Search Strategy

A comprehensive literature search was conducted across four electronic databases from inception through December 2025: PubMed/MEDLINE, Scopus, Web of Science, and Embase. The search strategy combined Medical Subject Headings (MeSH) terms with free-text keywords to maximize sensitivity.

The primary search combined terms for the index condition: (“oral lichen planus” OR “OLP” OR “lichen planus, oral”) AND (“differential diagnosis” OR “diagnosis” OR “diagnostic criteria” OR “histopathology”). Secondary searches targeted specific differential diagnoses: “oral lichenoid lesions” OR “lichenoid drug reaction” OR “lichenoid contact reaction”; “frictional keratosis” OR “traumatic keratosis” OR “benign alveolar ridge keratosis”; “chronic hyperplastic candidiasis” OR “candidal leukoplakia”; “oral leukoplakia” OR “proliferative verrucous leukoplakia”; (“lupus erythematosus” OR “discoid lupus”) AND “oral manifestations”; and “oral squamous cell carcinoma” AND “lichenoid features.”

Additional searches addressed diagnostic modalities and outcomes: “oral lichen planus” AND (“direct immunofluorescence” OR “immunofluorescence”); “oral lichen planus” AND (“malignant transformation” OR “oral squamous cell carcinoma” OR “cancer risk”); and “oral lichen planus” AND (“WHO criteria” OR “AAOMP” OR “diagnostic criteria”). Reference lists of included systematic reviews and key articles were manually screened to identify additional relevant studies.

### 2.3. Eligibility Criteria

Studies were included if they met the following criteria: English-language publication; focus on diagnosis, differential diagnosis, clinical features, histopathological characteristics, immunofluorescence patterns, or malignant transformation of OLP and related conditions; human subjects; and availability of full text. Eligible study designs included systematic reviews, meta-analyses, clinical practice guidelines, consensus statements, cohort studies, cross-sectional studies, and case series with ten or more patients.

Studies were excluded if they addressed exclusively therapeutic interventions without diagnostic content, comprised single case reports (except for the illustrative case from our institution), were published only as conference abstracts without subsequent full-text publication, or had been formally retracted.

### 2.4. Study Selection and Data Extraction

Given the narrative review design, formal dual independent screening was not performed. Instead, one author conducted the primary literature search and screening, with verification by a second author. Studies were selected based on relevance to diagnostic differentiation of OLP from mimicking lesions, methodological quality (prioritizing systematic reviews, meta-analyses, and large cohort studies), recency (with emphasis on publications from 2015 to 2025 to capture current evidence while including seminal earlier works), and clinical applicability for diagnostic decision-making.

Disagreements regarding study inclusion were resolved through discussion and consensus among the author team.

Data were extracted addressing the following domains: diagnostic criteria for OLP (clinical and histopathological); distinguishing features between OLP and differential diagnoses; direct immunofluorescence patterns and diagnostic performance metrics (sensitivity, specificity, odds ratios); malignant transformation rates with associated confidence intervals; risk factors for malignant transformation; and surveillance recommendations. Pooled estimates from meta-analyses were preferentially extracted; when unavailable, results from the largest or most methodologically rigorous individual studies were reported (Table 1).

### 2.5. Clinical Case Selection and Ethics

A representative clinical case from the Department of Odontology, Endodontics and Oral Pathology from Cluj-Napoca “Iuliu Hațieganu” University of Medicine and Pharmacy, Romania was selected to illustrate the practical application of the diagnostic framework. Case selection criteria included: presence of multiple differential diagnoses requiring systematic evaluation, coexistence of local traumatic factors complicating diagnosis, availability of histopathological confirmation, complete follow-up data demonstrating diagnostic and therapeutic outcomes, and patient consent.

The study was conducted in accordance with the Declaration of Helsinki. Institutional ethics committee approval was not required for this narrative review incorporating a single illustrative case, per local regulations. Written informed consent was obtained from the patient for publication of clinical details and accompanying non-identifiable images. The case is presented in accordance with the CARE (CAse REport) guidelines [21] to ensure completeness and transparency.

### 2.6. Data Synthesis

Evidence was synthesized qualitatively and organized thematically: evolution of diagnostic criteria, clinical and histopathological features of OLP, differential diagnosis categorized by etiology, role of ancillary testing, malignant transformation risk, and surveillance recommendations. A diagnostic algorithm was developed based on synthesized evidence and expert consensus among the authors.

### 2.7. Methodological Limitations

Consistent with the methodological characteristics of a narrative review, this study did not include prospective protocol registration, formal risk-of-bias assessment using standardized instruments, grading of the certainty of evidence, or quantitative meta-analysis. These limitations are inherent to the narrative approach, which emphasizes clinical synthesis and practical applicability rather than comprehensive systematic rigor. To minimize the impact of these limitations, we prioritized high-level evidence by focusing on existing systematic reviews and large-scale cohort studies to extract the recommendations. Furthermore, the inclusion of a detailed clinical case illustration bridges the gap between theoretical evidence and practical application, providing contextual nuance that standardized quantitative analyses often overlook. This dual approach ensures that while the review lacks statistical pooling, its clinical framework is grounded in validated diagnostic standards and real-world feasibility.

## 3. Results

### 3.1. Diagnostic Criteria for Oral Lichen Planus

The diagnosis of OLP has evolved significantly over five decades. The WHO published the first clinicopathological criteria in 1978, though these lacked consensus on clinical versus histological features and inadequately addressed the distinction from oral lichenoid lesions [1,2,22]. Modified WHO 2003 criteria improved differentiation, with studies using these criteria reporting lower malignant transformation rates (0.86%) compared to non-standardized criteria (1.01%) [1,3]. The AAOMP 2016 position paper further refined diagnostic standards, emphasizing mandatory clinicopathological correlation, continued follow-up as diagnosis may evolve, and recognition that OLP mimics may necessitate additional biopsies [4]. Inter-observer agreement using AAOMP criteria is good to very good, with clinicopathological correlation achieved in 87.6% of cases [6].

Clinically, OLP is characterized by a bilateral, often symmetrical presentation with Wickham striae—white, lacy, interlacing lines forming a reticular pattern [7,8,9,10]. The disease exhibits polymorphism (reticular, papular, plaque-like, erosive, atrophic, and bullous variants) with predilection for buccal mucosa (90%), gingiva, tongue, and palate. The reticular form predominates (40–70%), and presents as asymptomatic bilateral striae, whereas erosive/atrophic forms cause pain and functional impairment. The plaque-like variant carries significantly higher malignant transformation risk [11,23,24].

Histopathologically, classic OLP demonstrates hyperorthokeratosis or parakeratosis, “saw-tooth” rete ridges, basal cell liquefaction degeneration, and Civatte bodies (apoptotic keratinocytes) [4,7,9]. The hallmark is a dense, band-like lymphocytic infiltrate at the epithelial-connective tissue interface, predominantly T-lymphocytes with minimal eosinophils and plasma cells—the latter being significantly more numerous in oral lichenoid lesions [14,25].

Direct immunofluorescence (DIF) is not required for classic bilateral reticular OLP, but proves valuable for atypical presentations [1,4,26,27]. Oral Lichen Planus characteristically shows linear fibrinogen deposition at the basement membrane zone, with positive DIF significantly more likely in OLP versus oral lichenoid lesions [26,28]. Direct immunofluorescence differentiates autoimmune blistering diseases: pemphigus vulgaris shows intercellular IgG/C3 in a chicken-wire pattern; mucous membrane pemphigoid demonstrates linear IgG/C3 along the basement membrane zone (BMZ); and lupus erythematosus exhibits granular IgG/IgM/C3 (“lupus band”) [25,29,30,31].

### 3.2. Differential Diagnosis of Oral White Lesions

The differential diagnosis of OLP encompasses congenital white lesions, benign reactive lesions, infectious conditions, oral lichenoid lesions, autoimmune diseases, potentially malignant disorders, and malignancy [32].

White sponge nevus (Cannon’s disease) is the principal congenital lesion requiring differentiation from OLP. Key distinguishing features include an onset at birth or in early childhood, a positive family history, and the absence of symptoms or malignant potential. Histopathologically, white sponge nevus demonstrates hyperkeratosis with vacuolar changes in the spinous layer but lacks the band-like lymphocytic infiltrate and basal cell degeneration characteristic of OLP [10,33]. Recognition prevents unnecessary treatment of this benign condition.

Frictional keratosis presents as a white plaque at sites of chronic mechanical irritation—ill-fitting dentures, sharp teeth, or chronic cheek biting—with the key distinguishing feature being complete resolution after irritant removal [34,35]. Histopathology shows hyperparakeratosis and acanthosis but minimal inflammatory infiltrate, contrasting sharply with OLP. Benign alveolar ridge keratosis (BARK) is location-specific to edentulous ridges with “fading margins,” minimal inflammation, and very low dysplasia rate (2.1%) [34,36].

Chronic hyperplastic candidiasis presents as non-removable white plaques with rough surface texture, typically on the anterior buccal mucosa in patients with diabetes, denture wear, or immunosuppression [37,38]. Periodic Acid-Schiff (PAS)-positive fungal hyphae and neutrophilic microabscesses are diagnostic, and response to antifungal therapy distinguishes it from OLP. Notably, initial misdiagnosis as OLP occurs in 22.92% of cases, and malignant transformation rates reach 12.1% [38,39].

Oral lichenoid contact lesions show unilateral distribution with direct topographical relationship to dental restorations (commonly amalgam) and resolve after material removal [7]. Oral lichenoid drug reactions, associated with antihypertensives, oral hypoglycemics, NSAIDs, and antimalarials, present more erosively than OLP, with latency periods ranging from weeks to years [7]. Both demonstrate deeper perivascular inflammation and more prominent eosinophils/plasma cells than classic OLP [14,25,40,41].

Oral lupus erythematosus is distinguished by central erythema/atrophy with radiating peripheral striae (opposite to OLP’s pattern), perivascular inflammation, and granular DIF pattern (“lupus band”) [7,42]. Positive Antinuclear Antibody (ANA) and anti-double-stranded DNA (anti-dsDNA) support the diagnosis [43].

Leukoplakia—a diagnosis of exclusion—is typically unilateral, lacks the band-like lymphocytic infiltrate, and shows variable dysplasia [44]. Proliferative verrucous leukoplakia demonstrates progressive growth, verrucous texture, and carries >70% lifetime malignant transformation risk [34,45].

Oral squamous cell carcinoma (OSCC) with lichenoid features may mimic OLP but is distinguished by induration, unilateral presentation, rapid progression, and histological invasion [46]. Approximately 29% of dysplasia/carcinoma cases show ≥3 lichenoid features, with buccal mucosa overrepresented [46,47]. Importantly, 17.9% of OSCCs have OLP as a precursor, though OLP-associated OSCCs demonstrate a favorable prognosis: 5-year mortality 15.48% versus 34.70–50.00% for conventional OSCC [48,49,50].

### 3.3. Malignant Transformation

Pooled malignant transformation rates are 1.43% for OLP and 1.38% for oral lichenoid lesions, with an annual transformation rate of 0.2% [51,52]. Rates increase to 5.13% when epithelial dysplasia is present [52]. Diagnostic criteria significantly impact reported rates: modified WHO 2003 criteria yield 0.86% versus 1.01% for non-standardized criteria [3,53].

Risk factors include plaque-like subtype, tongue location, erosive/atrophic forms, tobacco use, alcohol consumption, combined tobacco/alcohol, and HCV infection [3,11,33,52,54,55,56]. Mean time from OLP diagnosis to OSCC ranges from 3.8 to 6.5 years, emphasizing the need for sustained surveillance [38,57].

### 3.4. Diagnostic Algorithm

Systematic evaluation should assess laterality (bilateral suggests OLP; unilateral suggests reactive lesions, lichenoid contact, or malignancy), pattern (reticular Wickham striae versus homogeneous plaque), location, symptoms, and progression (Table 2). 

Detailed history should capture medications, systemic diseases (HCV, diabetes, autoimmune conditions), tobacco/alcohol use, and dental restorations.

Local traumatic factors should be eliminated with 2–4 weeks of observation before biopsy. Mandatory biopsy indications include persistent lesions after irritant removal, unilateral presentation, erosive/ulcerative forms, induration, rapid growth, and high-risk sites (lateral/ventral tongue, floor of mouth). Histopathological examination should include PAS staining if candidiasis is suspected and dysplasia assessment. Direct immunofluorescence is indicated for atypical presentations, unilateral lesions, erosive/bullous forms, and diagnostic uncertainty [4,26,29].

Surveillance intervals should be every 6–12 months for standard OLP and every 3–4 months for high-risk features (erosive/atrophic, tongue location, plaque-like, tobacco/alcohol use) [2,9,10].

### 3.5. Clinical Case Illustration

An 81-year-old female non-smoker presented with oral discomfort and masticatory difficulty. Her medical history was notable for type 2 diabetes mellitus (Diaprel 60 mg/day), hypercholesterolemia (Roswera 5 mg/day), chronic ischemic heart disease (Nitromint 0.8 mg/day), essential hypertension requiring multiple antihypertensives (perindopril 10 mg/day, propranolol 120 mg/day, spironolactone/furosemide Diurex 50/20 mg/3 days), and osteoporosis—a constellation of comorbidities that would significantly influence both diagnostic reasoning and therapeutic decisions.

**Figure 1:**
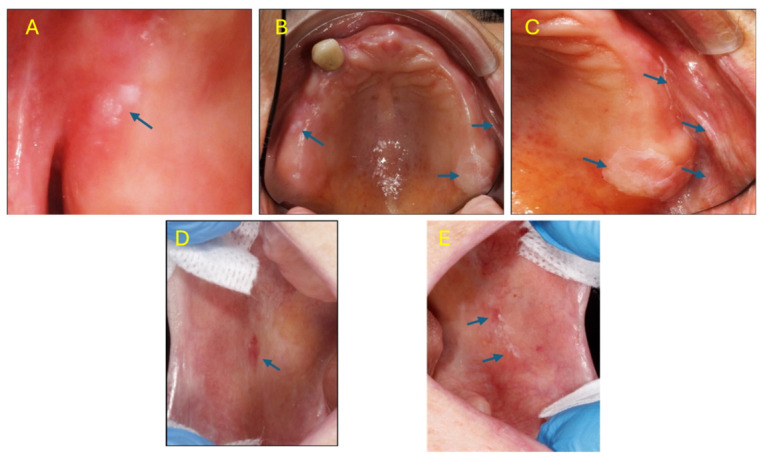
Identified lesions at the clinical examination. (**A**)—detailed image of irregular white lesion (arrow) located on the alveolar ridge, in the area of 1.7; (**B**)—overview of the hard palate and alveolar ridges (arrows); (**C**)—detailed image of the white plaque (arrows) located on the alveolar ridge, in the area of 2.7, 2.8, and its extension in the vestibule; (**D**)—fine lichenoid network and papules (arrow), with an erythematous area, located in the middle third of the right jugal mucosa; (**E**)—white plaque with lichenoid appearance, with small erythematous areas (arrows), at the level of the left jugal mucosa.

Intraoral examination revealed subtotal edentulism rehabilitated with poorly adapted partial removable acrylic dentures, demonstrating inadequate retention and compromised occlusion. Several remaining teeth showed advanced periodontal and prosthetic pathology, including failed endodontic treatments (on 1.3 and 3.3), deep cervical caries (3.3), poor marginal adaptation of crowns (1.3, 3.3, and 4.4), and grade 2/3 mobility (1.3 and 4.4). Multiple bilateral, non-scrapable white lesions with reticular and plaque-like patterns were identified: well-defined white plaques on the maxillary alveolar ridges (areas 1.7 and 2.7–2.8) extending to the vestibular mucosa, and bilateral buccal mucosal lesions with lichenoid appearance and small erythematous areas near the occlusal plane. All lesions were asymptomatic without erosion, ulceration, or induration. No extraoral or cutaneous involvement was present (Figure 1).

**Figure 2:**
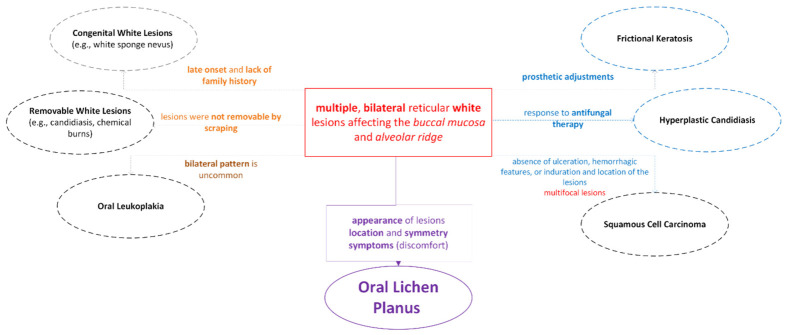
Differential Diagnosis Considerations based on Clinical Diagnostic Features: Ruling Out Reasoning.

The clinical presentation posed a diagnostic challenge requiring systematic evaluation. The bilateral, reticular buccal lesions strongly suggested OLP, yet the coexistence of extensive prosthetic trauma raised the possibility of frictional keratosis for the alveolar ridge lesions. The patient’s polypharmacy—particularly antihypertensives and oral hypoglycemics—introduced oral lichenoid drug reaction into the differential. Additionally, diabetes and denture wear constituted risk factors for chronic hyperplastic candidiasis, while squamous cell carcinoma could not be excluded for the plaque-like alveolar ridge lesions without histopathological confirmation (Figure 2).

A stepwise diagnostic approach was adopted. Initial management focused on eliminating local traumatic factors through temporary denture discontinuation, optimizing oral hygiene, and providing symptomatic topical therapy with chlorhexidine rinses and application of magisterial preparation twice a day: Neomycin 1 g, Streptomycin 1,000,000 IU, Metornidazole 500 mg, Hydrocortisone 25 mg, Xylin 2% 4 mL, Vitamin A 300,000 IU, Sodium bicarbonate 1 g, and glycerin 30 g. This conservative approach was justified by the absence of alarming features (no induration, rapid growth, or persistent ulceration) and the presence of clear mechanical irritants, with the expectation that short-term observation would differentiate reactive from persistent pathology.

**Figure 3:**
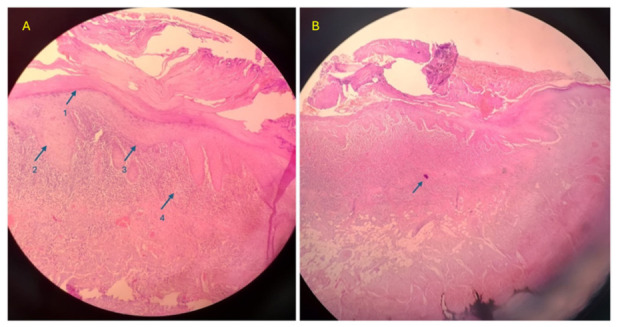
Histopathological examination: (**A**) 1—hyperkeratosis, 2—hypergranulosis, 3—degeneration of the basal layer, 4—band-like inflammatory infiltrate; (**B**) Civatte body (hematoxylin-eosin staining, magnification 40×).

At two-week follow-up, the patient reported partial improvement, but lesions persisted. A biopsy was performed from a persistent buccal mucosal lesion. Histopathological examination revealed classic OLP features: orthokeratotic hyperkeratosis, wedge-shaped hypergranulosis, basal layer liquefaction degeneration, “saw-tooth” rete ridges, band-like lymphocytic infiltrate masking the dermo-epidermal junction, and Civatte bodies (Figure 3). The diagnosis of oral lichen planus was confirmed.

Subsequent management included the extraction of non-restorable teeth and prosthetic rehabilitation with properly adapted dentures. Systemic corticosteroids were deliberately avoided given the patient’s osteoporosis, advanced age, and multiple comorbidities—illustrating the importance of individualized treatment planning. Prosthesis disinfection protocols and antifungal prophylaxis were instituted due to diabetes and denture wear.

**Figure 4:**
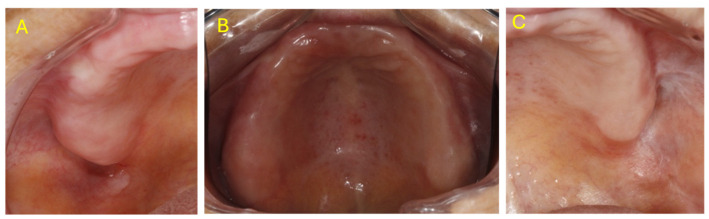
Three months follow-up examination: (**A**)—healthy alveolar ridge in the 1.7 area; (**B**)—overview of the hard palate and alveolar crest; (**C**)—healthy alveolar ridge in the 2.7, 2.8 area.

Three-month follow-up proved diagnostically illuminating: the alveolar ridge and vestibular lesions had completely resolved following prosthetic rehabilitation, confirming frictional keratosis, while the bilateral buccal mucosal lesions persisted with unchanged lichenoid appearance, consistent with OLP. No dysplasia or malignant transformation was observed (Figure 4).

This case illustrates several clinically relevant principles. First, multiple diagnoses can coexist—assuming a single etiology for all white lesions risks diagnostic error. Second, elimination of local factors before biopsy prevents unnecessary procedures and clarifies the clinical picture. Third, short-term observation (2–4 weeks) effectively differentiates reactive from persistent pathology. Fourth, polypharmacy complicates diagnosis, though bilateral symmetry and persistence despite continued medication use favored OLP over drug reaction in this case. Fifth, management must be individualized—systemic corticosteroids, though standard for symptomatic OLP, were appropriately avoided due to osteoporosis. Finally, despite asymptomatic presentation, long-term surveillance remains essential given OLP’s malignant potential.

## 4. Discussion

The presented synthesis of current evidence on distinguishing oral lichen planus from mimics of white lesions highlights key findings with direct implications for practice. The illustrated case demonstrated the efficacy of the systematic diagnostic approach, with initial management prioritizing the removal of local irritants and a period of clinical observation, subsequently followed by histopathological confirmation of oral lichen planus by biopsy in an 81-year-old case.

The main challenge remains the high variability in reported malignant transformation rates, which range from 0.86% with modified WHO 2003 criteria to over 1% with non-standardized definitions. This discrepancy is attributed to the historical misclassification of “lichenoid dysplasia” or reactive lichenoid lesions as true OLP. Our proposed framework addresses this variability by advocating for the strict clinicopathological correlation required by the AAOMP 2016 standards. By integrating a mandatory phase of local factor elimination—as demonstrated in our clinical case—clinicians can filter out reactive mimics (e.g., frictional keratosis) that often exhibit overlapping histological features. This ensures that surveillance and risk-assessment protocols are applied to a more accurately defined patient cohort, thereby aligning clinical expectations with the more specific, lower transformation rates found in contemporary standardized studies.

Diagnostic criteria have evolved substantially from WHO 1978 through modified WHO 2003 to the AAOMP 2016 position paper, with each iteration improving specificity [1,10]. The AAOMP criteria demonstrate good to very good inter-observer agreement when differentiating OLP from non-OLP conditions, with clinicopathological correlation achieved in 87.6% of cases [33]. While the AAOMP criteria improve inter-pathologist agreement, they are designed for cases in which OLP is already strongly suspected. In contrast to the AAOMP criteria, our framework acknowledges that in real-world practice, OLP often presents alongside local irritants or polypharmacy, which can confound even the strictest diagnostic criteria. By integrating a mandatory phase of local factor elimination—as seen in our patient—clinicians can identify reactive lesions (like frictional keratosis) that might otherwise be subjected to unnecessary biopsy or misdiagnosed as OLP due to overlapping histological features. This “stepwise logic” ensures that the AAOMP criteria are applied only to persistent, truly idiopathic lesions, thereby increasing their real-world specificity and clinical relevance. However, significant limitations persist in discriminating OLP from oral lichenoid lesions microscopically, underscoring the necessity of integrating clinical context with histopathological findings—a principle clearly demonstrated in our clinical case, where the bilateral symmetry and reticular pattern of buccal lesions provided essential diagnostic context that complemented histopathological confirmation [10,33].

Second, the differential diagnosis of OLP encompasses a broad spectrum of conditions—reactive (frictional keratosis, benign alveolar ridge keratosis), infectious (chronic hyperplastic candidiasis), lichenoid (contact and drug-induced reactions), autoimmune (lupus erythematosus), premalignant (leukoplakia, proliferative verrucous leukoplakia), and malignant (squamous cell carcinoma with lichenoid features) [7,44,58]. Our case exemplified this diagnostic complexity: the initial presentation included lesions on both buccal mucosae and alveolar ridges, requiring systematic consideration of OLP, frictional keratosis from ill-fitting prostheses, oral lichenoid drug reaction (given the patient’s antihypertensive and oral hypoglycemic medications), and exclusion of malignancy. Notably, approximately 29% of dysplastic and malignant lesions exhibit lichenoid histological features, emphasizing the critical importance of histopathological examination to exclude malignancy [44].

Third, direct immunofluorescence serves as a valuable adjunct in atypical presentations, demonstrating 73% sensitivity for OLP with characteristic linear fibrinogen deposition at the basement membrane zone. DIF effectively differentiates autoimmune blistering diseases based on distinct immunoglobulin patterns—intercellular IgG in pemphigus, linear IgG/C3 at the basement membrane in pemphigoid, and granular deposits in lupus erythematosus [1,42]. In our case, the classic bilateral reticular presentation did not necessitate DIF; however, had the lesions been unilateral or predominantly erosive, this ancillary test would have been indicated.

Fourth, malignant transformation rates of 1.43% for OLP (increasing to 5.13% with dysplasia) mandate long-term surveillance, particularly for high-risk subtypes including plaque-like lesions, tongue involvement, and erosive/atrophic forms [4,10]. Our patient’s lesions included plaque-like components, reinforcing the importance of regular follow-up despite the asymptomatic presentation.

### 4.1. Clinical Implications

The findings have several practical implications for clinicians evaluating oral white lesions. The applicability of the presented framework in complex scenarios is evidenced by its ability to resolve the inherent diagnostic overlap between OLP and lichenoid mimics. In cases involving oral lichenoid drug reactions or contact reactions, the histopathological features (such as band-like infiltrates) are often indistinguishable from idiopathic OLP. However, our framework introduces a “diagnostic through time” approach; by mandating the removal of local irritants and observing the clinical response over 2–4 weeks, clinicians can achieve a level of specificity that a static biopsy cannot provide. In our clinical case, the resolution of alveolar lesions following prosthetic adjustment confirmed a reactive etiology (frictional keratosis), while the persistence of buccal lesions confirmed OLP. This hierarchical approach minimizes the risk of diagnostic mimicry, ensuring that systemic or long-term treatments are only initiated for persistent, correctly identified OLP.

A systematic diagnostic approach—beginning with comprehensive clinical assessment, identification and elimination of local traumatic factors, short-term observation, selective biopsy, histopathological examination with ancillary testing when indicated, and clinicopathological correlation—optimizes diagnostic accuracy while avoiding unnecessary interventions. Our illustrative case demonstrated this approach in practice: the 81-year-old patient presented with multiple white lesions affecting both buccal mucosae and alveolar ridges. Rather than proceeding immediately to multiple biopsies, we first eliminated local traumatic factors (ill-fitting prostheses) and observed for 2–4 weeks. This strategy proved diagnostically valuable—alveolar ridge lesions resolved completely (confirming frictional keratosis), while persistent bilateral buccal lesions required biopsy, which confirmed OLP.

Multiple diagnoses may coexist, particularly in elderly individuals with comorbidities, polypharmacy, and local mechanical factors. The assumption that all white lesions share a single etiology may lead to diagnostic error. Our case powerfully illustrated this principle: the patient simultaneously had OLP (bilateral buccal mucosa, confirmed histopathologically) and frictional keratosis (alveolar ridges, confirmed by resolution after prosthetic rehabilitation). Assuming a single diagnosis, we might have either over-biopsied reactive lesions or, conversely, attributed all lesions to trauma and missed the OLP diagnosis.

Polypharmacy complicates differential diagnosis. The illustrated case was taking multiple medications associated with oral lichenoid drug reactions, including antihypertensives (perindopril, propranolol), diuretics (spironolactone/furosemide), and oral hypoglycemics (gliclazide), so drug-induced lichenoid lesions were possible [59,60]. However, several features favored OLP over drug reaction: bilateral symmetry, classic reticular pattern, and persistence despite continued medication use. In clinical practice, distinguishing OLP from oral lichenoid drug reactions remains challenging, particularly when medication discontinuation is not feasible due to medical necessity—as was the case for our patient’s cardiovascular and metabolic medications.

Treatment decisions must account for patient-specific factors, fitting individualized management. In our case, systemic corticosteroids—often first-line for symptomatic OLP—were appropriately avoided due to severe osteoporosis, advanced age (81 years), diabetes mellitus, and absence of erosive lesions. Instead, conservative management with topical therapy, oral hygiene optimization, and prosthetic rehabilitation achieved symptom resolution. This aligns with current recommendations emphasizing individualized risk-benefit assessment and highlights that not all OLP requires aggressive pharmacological intervention [61,62,63].

Given the malignant potential of OLP, regular follow-up is essential regardless of symptom status. Our patient’s lesions were asymptomatic and discovered incidentally during consultation for masticatory difficulties—underscoring that absence of symptoms does not negate the need for monitoring. Current evidence supports surveillance intervals of 6–12 months for standard OLP, with more frequent monitoring (every 3–4 months) for high-risk features [64,65,66].

Our findings align with and extend previous reviews on OLP diagnosis. Carrozzo et al. proposed that OLP and lichenoid lesions represent a spectrum of tissue reactions to unknown antigens, emphasizing the challenge of definitive categorization [7]. This concept resonates with our clinical case, where the distinction between OLP and oral lichenoid drug reaction could not be made with absolute certainty, given the patient’s polypharmacy—yet the bilateral symmetry and classic histopathological features favored OLP.

Müller highlighted that accurate diagnosis is often challenging due to overlapping clinical and histopathological features, and that dysplasia can exhibit lichenoid histology masking premalignant features [44]. Our review builds upon these observations by providing a structured diagnostic algorithm integrating clinical, histopathological, and immunofluorescence findings, illustrated through a real-world case demonstrating practical application.

The AAOMP position paper emphasized that OLP diagnosis requires clinicopathological correlation and continued follow-up, as the diagnosis may evolve over time [10]. Our clinical case reinforces this principle—initial presentation suggested multiple possible diagnoses, but systematic evaluation over three months enabled definitive differentiation between OLP and frictional keratosis. This temporal dimension of diagnosis is often underappreciated; our case demonstrates that patience and systematic follow-up can be as valuable for diagnosis as immediate intervention.

Novel diagnostic approaches, including cytokine profiling (elevated IFN-γ and IL-33 in OLP), salivary microbiome analysis, and CD123 immunohistochemistry for differentiating OLP from lupus erythematosus, have been explored [42,67,68]. A critical frontier in this research is the shift toward minimally invasive biomarkers that could supplement or, in specific monitoring scenarios, reduce the reliance on tissue biopsies. For instance, while CD123 and certain genetic polymorphisms require tissue samples or blood draws, salivary biomarkers and buccal swabs offer a non-invasive means of longitudinal monitoring. Salivary levels of pro-inflammatory cytokines and specific microRNA profiles are particularly promising as they reflect the local inflammatory environment of the oral mucosa without the morbidity of repeated biopsies [68]. However, the primary challenge remains the clinical validation of these non-invasive tools; specifically, determining whether a “liquid biopsy” can achieve the diagnostic specificity required to distinguish OLP from early-stage squamous cell carcinoma with the same reliability as histopathology. Until such validation is achieved, these approaches remain adjunctive rather than primary diagnostic modalities.

### 4.2. Strengths and Limitations

This review has several strengths. The PRISMA-inspired methodology ensured comprehensive and transparent evidence gathering while maintaining the flexibility necessary for clinical synthesis. Integration of a clinical case illustration provided a practical demonstration of systematic diagnostic reasoning in a complex patient—an elderly woman with multiple comorbidities, polypharmacy, and coexisting diagnoses—representing a scenario commonly encountered yet rarely addressed in the literature. The thematic organization offers clinicians a structured framework applicable to daily practice.

However, limitations must be acknowledged. As a narrative review, formal dual independent screening, risk of bias assessment, and quantitative meta-analysis were not conducted. The single illustrative case, while demonstrating key principles, cannot capture the full spectrum of diagnostic challenges seen in clinical practice. Additionally, the review focused on diagnosis rather than treatment, limiting therapeutic guidance beyond general principles.

### 4.3. Future Research Directions

Several areas warrant further investigation. First, standardization of diagnostic criteria remains incomplete—while the AAOMP criteria represent significant progress, inter-observer variability persists, particularly in distinguishing OLP from oral lichenoid lesions [33,69]. Development of more objective diagnostic markers would enhance reproducibility.

Second, novel diagnostic approaches show promise. Cytokine profiling, CD123 immunohistochemistry, and genetic polymorphism analysis may serve as adjunctive diagnostic tools but require validation [68,70,71]. While clinicopathological correlation remains the current diagnostic gold standard, future aspects of OLP management are shifting toward a more integrated molecular-digital paradigm. The reliance on subjective histopathological interpretation is expected to be supplemented by Artificial Intelligence (AI) algorithms capable of detecting subtle architectural changes in epithelial cells that may elude the human eye. Furthermore, the future of OLP diagnosis lies in molecular phenotyping—moving beyond the “white lesion” appearance to identify specific inflammatory endotypes via salivary proteomics or transcriptomics. This would allow a transition from a “one-size-fits-all” diagnostic category to personalized risk stratification, in which a patient’s unique molecular profile dictates the frequency of surveillance and the choice of therapeutic intervention. In due course, the future clinicopathological correlation will likely be a “multi-omic” correlation, integrating clinical morphology, digital pathology, and non-invasive molecular data.

Third, risk stratification for malignant transformation requires refinement. Current risk factors identify high-risk patients, but predictive accuracy remains suboptimal [49,72]. Molecular biomarkers identifying lesions at the highest transformation risk would enable targeted surveillance—particularly relevant for patients like ours with plaque-like components.

Finally, the relationship between OLP and systemic comorbidities—particularly diabetes, cardiovascular disease, and metabolic syndrome, all present in our case—merits further exploration, as these associations may inform both pathogenesis and management strategies [29].

## 5. Conclusions

Oral lichen planus diagnosis requires clinicopathological correlation—neither clinical nor histopathological assessment alone is sufficient. A systematic, stepwise approach—eliminating local traumatic factors, observing for resolution, performing selective biopsy of persistent lesions—optimizes diagnostic accuracy. The illustrated clinical case demonstrated this principle: short-term observation differentiated frictional keratosis (which resolved after prosthetic rehabilitation) from OLP (which persisted and was confirmed histopathologically).

Importantly, multiple diagnoses may coexist in the same patient, particularly elderly individuals with comorbidities and polypharmacy. Clinicians should avoid assuming all white lesions share a single etiology. Direct immunofluorescence serves as a valuable adjunct for atypical presentations but is not required for classic bilateral reticular OLP.

The malignant potential of OLP mandates long-term surveillance regardless of symptom status, with more frequent monitoring for high-risk features including plaque-like subtype, tongue location, and erosive/atrophic forms. Management must be individualized; conservative approaches may be appropriate for asymptomatic lesions in patients with contraindications to systemic therapy.

Future research should focus on standardizing diagnostic criteria, validating novel biomarkers, and refining the stratification of malignant transformation risk. Until such advances are realized, the structured diagnostic framework offers clinicians a practical, evidence-based approach to optimizing patient outcomes.

## Figures and Tables

**Table 1:** PRISMA-Inspired Flow Diagram for Literature Selection.

Category	Number of Articles/Studies
Records identified	214
Duplicates removed	58
Records screened	156
Records excluded	84
Full-text articles assessed for eligibility	72
Full-text articles excluded	21
Studies included in the present review	51
Studies included in the qualitative synthesis	29

**Table 2:** Summary of Differential Diagnosis of Oral White Lesions: Clinical Features, Histopathology, and Key Distinguishing Characteristics.

Condition	Clinical Features	Histopathology	Key Distinguishing Features
Oral Lichen Planus	Bilateral, symmetrical; reticular Wickham striae; buccal mucosa, gingiva, tongue.	Band-like lymphocytic infiltrate; basal cell degeneration; saw-tooth rete ridges; Civatte bodies.	Bilateral symmetry; reticular pattern; DIF: linear fibrinogen at BMZ.
White Sponge Nevus (Congenital)	Bilateral, white, thickened, spongy plaques; buccal mucosa; painless.	Hyperkeratosis; vacuolar changes in the spinous layer; normal basal layer; no inflammatory infiltrate.	Onset at birth/early childhood; positive family history; no malignant potential.
Frictional Keratosis	Unilateral white plaque at the site of trauma; identifiable irritant source.	Hyperparakeratosis; acanthosis; minimal inflammation; plasma pooling.	Resolves with irritant removal; lacks an inflammatory band.
Benign Alveolar Ridge Keratosis	White plaque on the edentulous ridge; fading margins; crestal location.	Hyperorthokeratosis; elongated rete pegs; minimal inflammation.	Location-specific (alveolar ridge only); no reticular pattern.
Chronic Hyperplastic Candidiasis	White plaque, rough surface; anterior buccal mucosa; cannot be wiped off.	Hyperparakeratosis with candidal hyphae (PAS+); neutrophilic microabscesses.	PAS-positive hyphae; responds to antifungal therapy.
Oral Lichenoid Contact Lesion	Unilateral; corresponds to dental restoration; resolves with material removal.	Similar to OLP; deeper perivascular inflammation; more eosinophils/plasma cells.	Topographical relationship to restoration; unilateral.
Oral Lichenoid Drug Reaction	Bilateral or unilateral; erosive > reticular; temporal relationship with medication.	Similar to OLP; more eosinophils/plasma cells; deeper inflammation.	Improves with drug cessation; more erosive presentation.
Oral Lupus Erythematosus	Central erythema/atrophy with radiating striae; skin involvement (butterfly rash).	Perivascular/periappendageal inflammation; thickened BMZ (PAS+).	DIF: granular IgG/IgM/C3; positive ANA; skin lesions.
Leukoplakia	Unilateral white plaque; homogeneous or non-homogeneous; diagnosis of exclusion.	Hyperkeratosis; variable dysplasia; lacks an inflammatory band.	Unilateral; no reticular pattern; no basal degeneration.
Proliferative Verrucous Leukoplakia	Multifocal or >4 cm; progressive growth; verrucous surface.	Corrugated hyperorthokeratosis; verrucous architecture; progressive dysplasia.	Progressive growth; verrucous texture; very high malignancy risk (>70%).
Oral Squamous Cell Carcinoma	Often unilateral; persistent ulceration or induration, rapid growth; may show lichenoid margins.	Malignant epithelial cells; basement membrane invasion; nuclear pleomorphism; disordered maturation.	Unilateral; induration (hardness); rapid progression; histological evidence of invasion.

## Data Availability

No new data were created or analyzed in this study.
